# Cytokine-Induced Killer Cells in Combination with Heat Shock Protein 90 Inhibitors Functioning via the Fas/FasL Axis Provides Rationale for a Potential Clinical Benefit in Burkitt’s lymphoma

**DOI:** 10.3390/ijms241512476

**Published:** 2023-08-05

**Authors:** Fangfang Ge, Yulu Wang, Amit Sharma, Yu Yang, Hongde Liu, Markus Essler, Ulrich Jaehde, Ingo G. H. Schmidt-Wolf

**Affiliations:** 1 Department of Integrated Oncology, Center for Integrated Oncology (CIO), University Hospital of Bonn, 53127 Bonn, Germany; gefangfang3@gmail.com (F.G.); yuluwang3@gmail.com (Y.W.); amit.sharma@ukbonn.de (A.S.); 2 Department of Neurosurgery, University Hospital Bonn, 53127 Bonn, Germany; 3 State Key Laboratory of Bioelectronics, School of Biological Science & Medical Engineering, Southeast University, Nanjing 210096, China; 220212221@seu.edu.cn (Y.Y.); liuhongde@seu.edu.cn (H.L.); 4 Department of Nuclear Medicine, University Hospital Bonn, 53127 Bonn, Germany; markus.essler@ukbonn.de; 5 Department of Clinical Pharmacy, Institute of Pharmacy, University of Bonn, 53121 Bonn, Germany; u.jaehde@uni-bonn.de

**Keywords:** CIK cell, HSP90 inhibitor, Burkitt’s lymphoma, Fas–FasL, apoptosis

## Abstract

Constant efforts are being made to develop methods for improving cancer immunotherapy, including cytokine-induced killer (CIK) cell therapy. Numerous heat shock protein (HSP) 90 inhibitors have been assessed for antitumor efficacy in preclinical and clinical trials, highlighting their individual prospects for targeted cancer therapy. Therefore, we tested the compatibility of CIK cells with HSP90 inhibitors using Burkitt’s lymphoma (BL) cells. Our analysis revealed that CIK cytotoxicity in BL cells was augmented in combination with independent HSP90 inhibitors 17-DMAG (17-dimethylaminoethylamino-17-demethoxygeldanamycin) and ganetespib. Interestingly, CIK cell cytotoxicity did not diminish after blocking with NKG2D (natural killer group 2, member D), which is a prerequisite for their activation. Subsequent analyses revealed that the increased expression of Fas on the surface of BL cells, which induces caspase 3/7-dependent apoptosis, may account for this effect. Thus, we provide evidence that CIK cells, either alone or in combination with HSP90 inhibitors, target BL cells via the Fas–FasL axis rather than the NKG2D pathway. In the context of clinical relevance, we also found that high expression of HSP90 family genes (*HSP90AA1*, *HSP90AB1*, and *HSP90B1*) was significantly associated with the reduced overall survival of BL patients. In addition to HSP90, genes belonging to the Hsp40, Hsp70, and Hsp110 families have also been found to be clinically significant for BL survival. Taken together, the combinatorial therapy of CIK cells with HSP90 inhibitors has the potential to provide clinical benefits to patients with BL.

## 1. Introduction

Despite having an unprecedented understanding of cancer today [1,2,3], it remains one of the leading causes of death. Certainly, there have been a number of efforts to improve treatment options for cancer, most notably by exploring various inhibitors (most recently HSP90 C-terminal inhibitors) in order to understand the molecular and cellular determinants harboring any clinical relevance [4,5,6]. Since it has been recognized that the overexpression of HSP90 plays a potential role in the development of cancer, researchers have begun to modulate its activity to combat cancer [7,8,9]. Interestingly, HSP90 overexpression has been observed in numerous cancers, including breast, urinary, ovarian, lung, colon, esophageal, ovarian, endometrial, bone, and prostate cancers [10,11]. HSP90 encompasses various isoforms that exhibit distinct accumulation patterns within cellular compartments. Among the prominent cytoplasmic variants, *HSP90AA1* and *HSP90AB1* have been observed to not only localize in the cytoplasm but also in the nucleus. On the other hand, HSP90B1-GRP94 predominantly targets the endoplasmic reticulum lumen, where it plays a role in the unfolded protein response [12]. Another significant HSP90 isoform, TNF-receptor-associated protein-1 (TRAP1), primarily functions as a mitochondrial protein, contributing to mitochondrial integrity, apoptosis regulation, and protection against oxidative stress [13,14]. *HSP90AA1*, *HSP90AB1*, and *HSP90B1* have been associated with a poor prognosis of tumors [15], and upregulation of TRAP1 promotes the growth and progression of various cancers [16].

Burkitt’s lymphoma (BL), a rare but extremely aggressive B-cell non-Hodgkin’s lymphoma that is usually diagnosed in children and adolescents, seems to be somewhat unique. Valbuena et al. reported moderate to strong cytoplasmic expression levels of HSP90 in all BL cases [17]. Giulino-Roth et al. reported that primary BL tumors overexpress HSP90, and that its inhibition exerts antitumor effects both in vitro and in vivo [18]. These authors found that the inhibition of HSP90 targets multiple components of PI3K/AKT/mTOR signaling, underscoring the importance of this pathway in BL. By characterizing the molecular consequences of HSP90 inhibition in BL cells, Walter et al. provided evidence that SYK is a client protein of HSP90, and that the BCR signaling-dependent phosphorylation of Hsp90 is required for this interaction [19]. Based on these findings, Poole et al. speculated that the MYC-HSP90 axis may be critical for tumor maintenance in BL and may represent a novel therapeutic strategy [20]. These authors showed that the MYC oncogene is a client protein of HSP90 in BL, and that its inhibition using pharmacological inhibitors causes MYC transcription switching and protein destabilization. Overall, HSP90 appears to be a critical component of BL; however, chemotherapy remains the mainstay of BL treatment in the clinic. The use of immunotherapies for BL has also been reported [21,22]; however, their success rates have not been fully elucidated. Notably, the success rates in refractory/relapsed BL cases are extremely low; therefore, there is an urgent need to identify new options. Strikingly, cytokine-induced killer (CIK) cell therapy, which has been successful for 30 years in treating various cancers, has never been tested for BL [23,24,25,26].

Being a mixture of cells, CIK cells include T cells (CD3+CD56-), NKT cells (CD3+CD56+), and NK cells (CD3-CD56+). CIK cells can significantly lyze cancer cells in an MHC-unrestricted manner by activating NK cell receptors [27]. Alongside directly killing tumor cells, CIK cells can also regulate immune function by releasing various cytokines. Evidence has shown that after stimulation by tumor cells, the amount of proinflammatory cytokines, such as tumor necrosis factor (TNF)-α, IFN-γ, and IL-2, released by CIK cells were significantly increased [28], and these cytokines potentiate systemic antitumor activity. The first clinical trial of CIK cells in lymphoma was reported by Schmidt-Wolf et al. in 1999 [29]. Subsequently, 17 clinical trials have been conducted for the treatment of lymphoma owing to the proven safety of CIK cell therapy [30].

Therefore, in this study, we assessed the compatibility of CIK cells with HSP90 inhibitors (17-DMAG and ganetespib) in diverse BL cell lines. In addition to highlighting the possible mechanism behind their favorable synergistic effect, we discussed the clinical significance of the expression patterns of the HSP90 family (including *HSP90AA1*, *HSP90AB1*, and *HSP90B1*) and nine other heat shock proteins (HSPs) in the survival of patients with BL. To our knowledge, this is the first study to use foreground CIK cell therapy as a potential treatment option for patients with BL.

## 2. Results

### 2.1. HSP90 Inhibitors Showed Synergistic Effect with CIK Cells in Burkitt’s Lymphoma Cells

**Figure 1:**
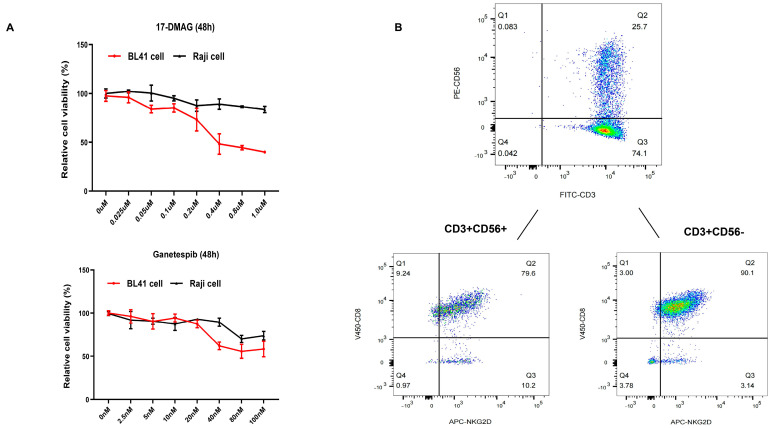
(**A**) Cell viability of Burkitt’s lymphoma (BL) cells was assessed following treatment with HSP90 inhibitors. Subsequently, the optimal concentration of HSP90 inhibitors was determined (17-DMAG-0.1 µM and ganetespib-20 µM, respectively). The presented data represent the mean ± standard deviation (SD) of triplicates per experimental condition. (**B**) Phenotype of day 14 CIK cells. The figure shows data from one representative from four donors.

**Figure 2:**
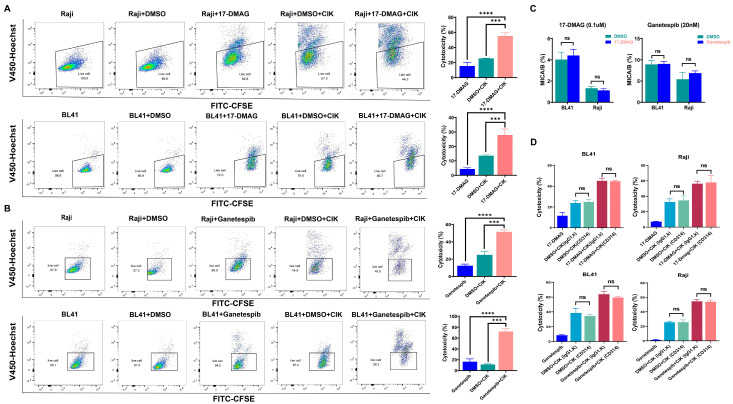
Cytotoxicity of CIK cells treated with/without 17-DMAG (**A**) and ganetespib (**B**) against Raji and BL41 cells. Analyses were performed on different donors (*n* = 3) and values are expressed as mean ± SD. Analyses were performed using the Student’s *t*-test. (**C**) MICA/B expression on the surface of Raji and BL41 cells after treatment with 17-DMAG and ganetespib. Each bar represents the mean ± SD of a representative donor. (**D**) Cytotoxicity of CIK cells treated with/without two HSP90 inhibitors against Burkitt’s lymphoma cell lines with/without NKG2D blocking (CIK cells were completely blocked before co-culture with Raji and BL41 cells). Cytotoxicity assays were conducted on CIK cells between days 14 and 16. CIK cells and BL cell lines were co-cultured with or without HSP90 inhibitors for 24 h at an E/T ratio of 10:1. Analyses were performed on 3 donors and one of them has been indicated. Data analysis was performed using the one-way ANOVA. *** *p* < 0.001, **** *p* < 0.0001, ns: not significant.

First, we investigated the viability of BL cells (specifically BL41 and Raji cells) using two independent HSP90 inhibitors (17-DMAG and ganetespib). An optimal concentration of 17-DMAG (0.1 µM) and ganetespib (20 nM) was used for further experiments based on the co-culture experiments (Figure 1A). Thereafter, the subtypes of CIK cells were examined (day 14), and NKT cells (CD3+CD56+, 27.66% ± 4.573%), T cells (CD3+CD56-, 71.85% ± 4.607%), and NK cells (CD3-CD56+, 0.7% ± 0.2%) were identified (Figure 1B). Considering that NKG2D and CD8 were mainly expressed on NKT and T cells, we also examined their individual proportions, as shown in Figure 1B. In the context of synergy, significant results were obtained with Raji cells, while the combined effect of DMSO with CIK cells (25.46% ± 0.2301%, *p* = 0.0002) and the isolated 17-DMAG (15.38% ± 5.007%, *p* < 0.0001) treatment group demonstrated a lower efficacy compared to the combination of CIK cells with 17-DMAG (55.25% ± 4.725%) (Figure 2A). Similar results, especially in BL41 cells, were obtained in that the cytotoxicity of CIK cells in combination with 17-DMAG (27.91% ± 4.176%) was significantly higher than that of the controls, such as the DMSO in combination with CIK cells (13.6% ± 0.7162%, *p* = 0.0008) and isolated 17-DMAG (4.317% ± 0.8552%, *p* < 0.0001) (Figure 2A) groups. In addition, the above findings were confirmed with ganetespib, as the results showed that ganetespib in combination with CIK cells (51.47% ± 2.357%) significantly increased the cytotoxicity against Raji cells compared to the controls, ganetespib alone (12.33% ± 1.911%, *p* < 0.0001), and DMSO in combination with CIK cells (24.90% ± 3.954%, *p* < 0.0001) (Figure 2B). Similarly, for BL41 cells, the cytotoxicity of CIK cells in combination with ganetespib (72.12% ± 5.537%) was higher than that of the controls, isolated ganetespib (16.37% ± 5.272, *p* < 0.0001) treatment, and DMSO in combination with CIK cells (11.19 ± 1.295, *p* < 0.0001) (Figure 2B). This indicates that both HSP90 inhibitors, 17-DMAG and ganetespib, have synergistic effects with CIK cells against BL cell lines.

### 2.2. Cytotoxic Effect of CIK Cells Synergizing with HSP90 Is Independent of the NKD2D/NKG2DL Axis

Given that the NKG2D/NKG2DL axis plays an important role in the anticancer properties of CIK cells, MICA/B is the major NKG2D ligand on the surface of tumors. Therefore, we evaluated MICA/B expression on the surface of BL cells (Raji and BL-41) and investigated any possible alterations induced by HSP90 inhibitors (Figure 2C). Interestingly, we found that BL cells treated with 17-DMAG and ganetespib did not show altered MICA/B expression levels compared to the controls. Moreover, no effect on the cytotoxicity of CIK cells was observed when an anti-human CD314 (NKG2D) antibody was used to block the NKG2D/NKG2DL axis between the CIK and BL cells (Figure 2D). Importantly, we observed similar effects similar to those of ganetespib (Figure 2D). This suggests that the NKD2D/NKG2DL axis does not contribute to the cytotoxic effect of CIK cells in synergy with HSP90 inhibitors in BL.

### 2.3. CIK Cells Combined with HSP90 Inhibitors Primarily Induced Apoptosis in BL

**Figure 3:**
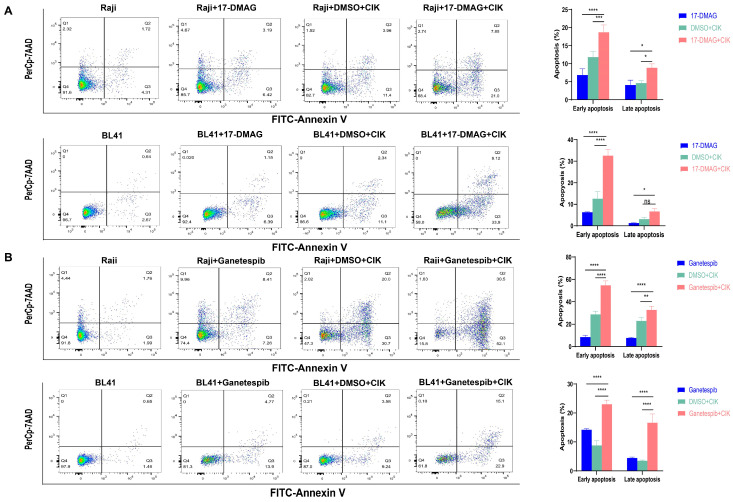
Apoptosis in Raji and BL 41 cells co-cultured with/without CIK cells treated with 17-DMAG (**A**) and ganetespib (**B**). In the apoptosis experiment involving BL cell lines, we utilized CIK cells at days 14–16, co-cultured with or without HSP90 inhibitors for 24 h, with an E/T ratio of 5:1. Each bar represents the mean ± SD of a triplicate measurement, and these data are representative of three independent experiments. Data analysis was conducted using the two-way ANOVA. * *p* < 0.05, ** *p* < 0.01, *** *p* < 0.001, **** *p* < 0.0001, ns: not significant.

Next, we investigated the possible activation of apoptosis induced by CIK cells and/or HSP90 inhibitors in BL cells. It was found that early apoptosis significantly increased in Raji cells and 17-DMAG combined with CIK cells (18.767% ± 1.966%) compared with 17-DMAG (6.893% ± 1.7%, *p* < 0.0001) and CIK cells (11.867% ± 1.457%, *p* = 0.0003) (Figure 3A). Interestingly, equally significant results were found for BL41 cells when 17-DMAG was combined with CIK cells (32.667% ± 2.857%) compared to the control groups, 17-DMAG (6.343% ± 0.184%, *p* < 0.0001), and CIK cells (12.67% ± 3.247%, *p* < 0.0001) (Figure 3A). As a proof of concept, we also tested ganetespib and found comparable results to 17-DMAG. In Raji cells, an increase in early apoptosis was observed when ganetespib was co-cultured with CIK cells (54.8% ± 3.818%), compared with ganetespib alone (8.533% ± 1.443%, *p* < 0.0001) and CIK cells (28.85% ± 2.616%, *p* < 0.0001) (Figure 3B). Ganetespib and CIK cells (23.067% ± 1.358%) also exhibited increased early apoptosis in BL cells compared to ganetespib alone (14.233% ± 0.351%, *p* < 0.0001) and CIK cells (8.833% ± 1.609%, *p* < 0.0001) (Figure 3B). Thus, the combination of HSP90 inhibitors with CIK cells has demonstrated the ability to enhance late apoptosis in Burkitt’s lymphoma (BL), particularly with ganetespib. In Raji cells, a notable increase in late apoptosis was observed when ganetespib was combined with CIK cells (32.75 ± 3.182), compared to the individual treatments of ganetespib (7.857 ± 0.49, *p* < 0.0001) and CIK cells (23.167 ± 2.747, *p* = 0.0052) (Figure 3B). Similarly, significant results were obtained with BL41 cells, where the combination of ganetespib and CIK cells (16.7% ± 2.946%) demonstrated a substantial increase in late apoptosis compared to the control groups, ganetespib alone (4.5% ± 0.298, *p* < 0.0001), and CIK cells alone (3.54% ± 0.233%, *p* < 0.0001) (Figure 3B). Thus, CIK cells, in combination with HSP90 inhibitors, were responsible for inducing early apoptosis in BL cells.

### 2.4. Increased Expression of Fas May Lead to the Induction of Caspase 3/7-Dependent Apoptosis in BL Cells via HSP90 Inhibitors

**Figure 4:**
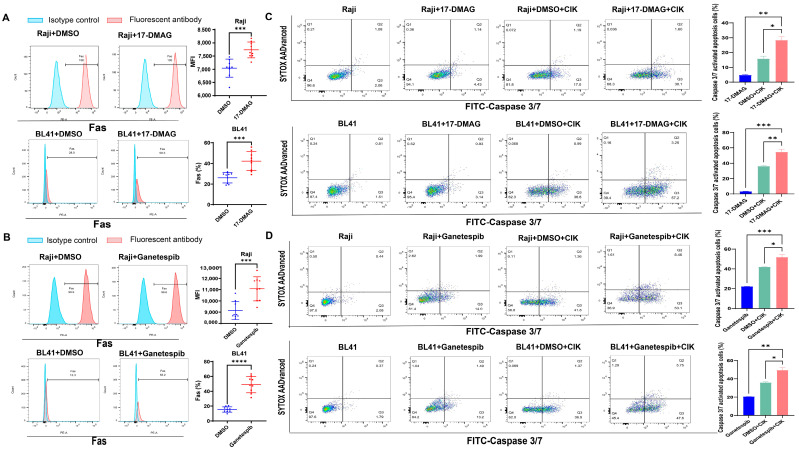
Fas expression on the surface of BL41 and Raji cells treated with/without 17-DMAG (**A**) and ganetespib (**B**). Fas expression was measured using flow cytometry following treatment with or without HSP90 inhibitors for 48 h. Each bar represents the mean ± SD of three independent experiments. Data analysis was conducted using the Student’s *t*-test. Caspase 3/7-activated apoptotic cells on BL41 and Raji cells treated with/without the 17-DMAG (**C**) and ganetespib (**D**) combination of CIK cells for 12 h, with an E/T of 2.5:1. These data are representative of three independent experiments and analysis was performed using the one-way ANOVA. * *p* < 0.05, ** *p* < 0.01, *** *p* < 0.001, **** *p* < 0.0001.

In addition to NKG2D/NKG2D, Fas/FasL is considered an alternative pathway for the cytotoxicity of CIK cells, leading to apoptosis; therefore, we examined Fas expression on the surfaces of BL cells. Interestingly, we found that after treatment with 17-DMAG, the percentage of Fas expression in BL41 cells significantly increased (*p* = 0.0003) (Figure 4A). After confirming that Fas was expressed in almost all Raji cells, we then assessed the MFI (mean fluorescence intensity) values and confirmed the significantly increased effect of 17-DMAG (*p* = 0.0426) (Figure 4A). Similar results were observed when ganetespib was administered (as shown in Figure 4B). Notably, a significant increase in caspase-3/7-activated apoptotic cells was observed in BL41 and Raji cells when CIK cells were combined with 17-DMAG compared to 17-DMAG (*p* = 0.0004 and *p* = 0.002, respectively) and CIK cells (*p* = 0.0088 and *p* = 0.0123, respectively) alone (Figure 4C). Ganetespib, in co-culture with CIK cells, also supported the increase in caspase-3/7-activated apoptotic cells in BL41 and Raji cells compared to the ganetespib (*p* = 0.0012 and *p* = 0.0008, respectively) and CIK cell groups (*p* = 0.0112 and *p* = 0.0191, respectively) (Figure 4D). This suggests that the increased expression of Fas on the BL cell surface may lead to caspase 3/7-dependent apoptosis induction via HSP90 inhibitors.

### 2.5. High Expression of HSP90 Genes Is Significantly Associated with Reduced Overall Survival in BL Patients

**Figure 5:**
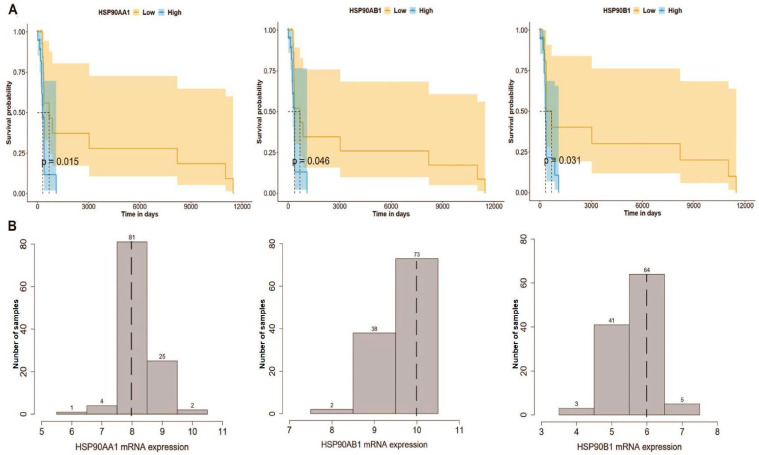
Expression of three HSP90 genes (*HSP90AA1*, *HSP90AB1*, and *HSP90B1*) predicts the survival of Burkitt’s lymphoma patients. (**A**) Kaplan–Meier survival curve based on overall survival based on gene expression in a cohort of TCGA Burkitt’s lymphoma patients, 95% confidence interval and the *p*-value (logarithmic rank test, Mantel–Haenszel). (**B**) Distribution of three HSP90s expression in the TCGA Burkitt’s lymphoma dataset. The dotted lines indicate the median gene expression used as a cutoff to normalize the mRNA data, which were then log2 processed.

**Figure 6:**
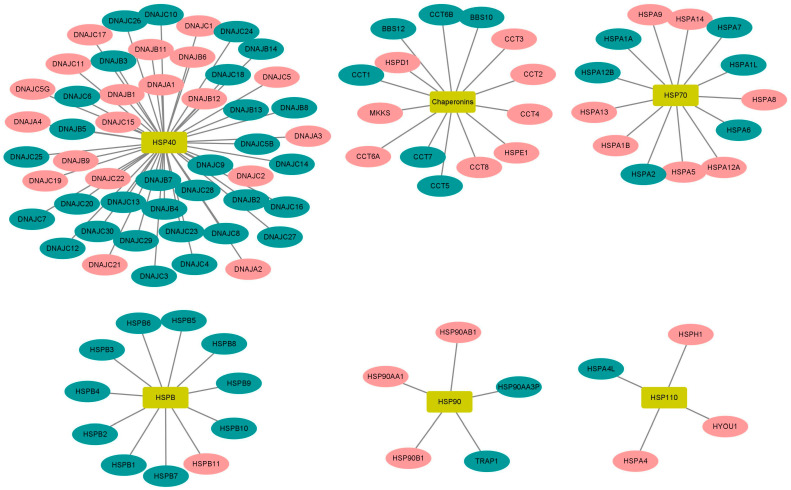
Association of 96 genes encoding heat shock proteins with overall survival of Burkitt’s lymphoma patients from the KM plotter. The red color represents the genes that are related with survival, while the green color denotes the genes that are not related with survival.

Considering that HSP90 is not the only HSP involved in cancer, we next investigated the prognostic ability of HSP90 and other HSP-related genes (*n* = 84) using the TCGA dataset for BL. We first performed a log-rank test (Mantel–Haenszel) to detect significant differences in patient survival depending on HSP gene expression (low and high expression groups). Importantly, we found that the high expression of HSP90 genes (namely *HSP90AA1*, *HSP90AB1*, and *HSP90B1*) was significantly associated with reduced overall survival in the KM-plotter cohort (*p* = 0.015; *p* = 0.046; and *p* = 0.031, respectively) (Figure 5). In addition to the HSP90 genes, several other HSP genes, including *HSPA1B*, *HSPA4*, *HSPA9*, *HSPA14*, *HYOU1*, *HSPB11*, *MKKS*, *DNAJA1*, *DNAJA3*, *DNAJA4*, *DNAJB6*, *DNAJB9*, *DNAJB11*, *DNAJC2*, *DNAJC15*, *DNAJC17*, *DNAJC19*, and *CCT8* were also found to be clinically significant for BL survival (*p* ≤ 0.05) (Figure 6 and Supplementary Table S1). Thus, these findings strengthen the importance of our preclinical model (CIK cells and HSP90) for BL and prompts a more in-depth investigation of other HSP candidates.

## 3. Discussion

Although cancer immunotherapy has become the mainstay of cancer treatment, a minority of cancer patients still do not respond to it. Consequently, researchers and clinicians are currently identifying and investigating alternative and combined treatment modalities. Immune checkpoint inhibitors (ICI) have been very successful in this context, but the ability of heat shock proteins (HSPs), especially HSP90, to trigger the immune system against tumor cells has also been of particular interest. HSP90 inhibitors have been successfully assessed across a wide range of preclinical/clinical cancers, including gliomas [31], breast cancer [32], lung cancer [33], prostate cancer [34], and colorectal cancer [35]. Furthermore, HSP90 has been implicated in hematological malignancies, including acute myeloid leukemia [36], mantle cell lymphoma [37], acute lymphoblastic leukemia [38], T-cell leukemia [39], and chronic lymphocytic leukemia [40]. Interestingly, the outcomes of clinical trials involving HSP90 in various cancer types (including hematological malignancies) have been encouraging [41,42,43,44,45,46]. Surprisingly, HSP90 has never been tested with cytokine-induced killer (CIK) cell therapy, which has recently turned 30 years old and has been shown to be successful across various hematological malignancies, including B-cell malignancies [47], multiple myeloma [48], and acute myeloid leukemia [49]. Considering that Burkitt’s lymphoma (BL) continues to be a challenging hematological malignancy, we investigated whether the synergy of HSP90 and CIK cells could pave the way for a potential treatment. To widen our analysis, we used both EBV-negative (BL41) and EBV-positive (Raji) cell lines in this first preclinical study.

To address this, we first checked the viability of BL cells (BL41 and Raji) using two independent HSP90 inhibitors (17-DMAG and ganetespib) with CIK cells and confirmed a synergistic effect in all combinations. It has been well established that the NKG2D/NKG2DL axis plays an important role in the anticancer properties of CIK cells, and MICA/B is the major NKG2D ligand on the surface of tumors [50]. Next, we assessed MICA/B expression on the surface of BL cells and evaluated possible changes induced by HSP90 inhibitors. The results clearly showed that neither HSP90 inhibitor had an effect on MICA/B expression levels in BL cells. In addition, when anti-human CD314 (NKG2D) antibody was used to block the NKG2D/NKG2DL axis between the CIK cells and BL cells, no effect was apparent. In particular, CIK cells, in combination with HSP90 inhibitors, induced early apoptosis in BL cells. A study on the combination therapy of HSP90 inhibitors for colorectal cancer indicated that HSP90 can enhance immunotherapy through the Fas/FasL axis between cancer cells and T cells, HSP90 was able to improve immunotherapy through cancer cells and the T cell Fas/FasL axis [35]. It was further evident that the increased expression of Fas on the surface of BL cells resulted in the induction of caspase 3/7-dependent apoptosis. Thus, it is possible that the Fas/FasL pathway, rather than the NKG2D/NKG2D pathway, serves as an alternative mechanism for CIK cell cytotoxicity (with HSP90) in BL.

Using TCGA datasets for BL, we examined the prognostic ability of HSP90 and found that high expression levels of the HSP90 family (specifically *HSP90AA1*, *HSP90AB1*, and *HSP90B1*) were significantly associated with reduced overall survival. This suggests that the HSP90-CIK cell combination may serve as an alternative treatment strategy for BL. However, it cannot be excluded that other HSP-related genes (in addition to HSP90) may also have an importance in BL. Therefore, we evaluated the expression of all HSP-related genes (*n* = 84), and nine of them, including *HSPA1B*, *HSPA8*, *HSPB11*, *DNAJA3*, *DNAJB9*, *DNAJC11*, *DNAJC17*, *DNAJC19*, and *DNAJC22*, were found to be clinically significant for BL survival. Importantly, a few of them have already been implicated in different cancers, such as AML [51], gastric cancer [52], colon cancer [53], lung cancer [54,55], liver cancer [56], B-cell lymphoma [57] and breast cancer [58]. Taken together, HSPs are closely associated with BL, and their suitability for CIK cells has been warranted.

It is also worth mentioning that we used first-generation (17-DMAG) and second-generation (ganetespib) HSP90 inhibitors as a combination strategy with CIK cells, and the future availability of dual inhibitors (such as HSP90-HDAC [59]) may further contribute to defining the cytotoxic efficacy of CIK cells in BL. The cytotoxic ability of CIK cells has been successfully demonstrated in several clinical studies. The functional aspect of the cytotoxicity of CIK cells via the NKG2D/NKG2DL signaling pathway and/or Fas/FasL signaling has also been widely discussed in the literature. However, the exact and alternative modes of functioning for these cells is still unclear, and the involvement of other mechanisms cannot be ruled out. Particularly, in the context of BL, our study is the first attempt to show that HSP90 inhibitors can enhance the cytotoxic effect of CIK cells via the Fas/FasL signaling pathway, which consequently activates caspase3/7-dependent apoptosis. Certainly, further experiments, especially in vivo, can help to gain further insight into the deeper mechanisms, but at the preclinical level, our study is the first attempt to investigate the suitability of cancer immunotherapy of CIK cells together with HSP90 in Burkitt’s lymphoma.

## 4. Materials and Methods

### 4.1. Cell Lines and Cell Culture

The Burkitt’s lymphoma lines (Raji and BL41) were cultured in RPMI-1640 (Pan-Biotech, Aidenbach, Bavaria, Germany) medium supplemented with 10% FBS (Sigma-Aldrich Chemie GmbH, Munich, Germany) and 1% penicillin/streptomycin (P/S) (Gibco, Schwerte, Germany) at 37 °C and 5% CO_2_. Both cell lines were purchased from DSMZ (Braunschweig, Germany) and detected as mycoplasma-free using a Mycoplasma Detection Kit (Thermo Fisher Scientific, Darmstadt, Germany).

### 4.2. Reagents

The antibodies (FITC-CD3, PE-CD56, APC-NKG2D, BV421-CD8, and APC-MICA/B) and their respective isotype antibodies were purchased from BioLegend (San Diego, CA, USA). The Annexin V-FITC -7AAD (7-Amino-Actinomycin D) kit was also purchased from BioLegend (San Diego, CA, USA). The HSP90 inhibitors 17-DMAG and ganetespib (STA-9090) were purchased from Selleckchem (Boston, MA, USA). These HSP90 inhibitors were dissolved in DMSO and stored at −80 °C at a concentration of 50 mM (please note that the control DMSO concentration of 17-DMAG was 0.1‰, and that the control DMSO concentration of ganetespib was 0.02‰). The FxCycle™ Violet stain and CellTrace™ CFSE Cell Proliferation Kit (Invitrogen, Thermo Fisher Scientific, Eugene, OR, USA) were used to distinguish tumor cells from CIK cells using flow cytometry. Hoechst 34580 (Merck, Sigma, Darmstadt, Germany) was added prior to flow cytometry to stain the dead cells. The CellEvent Tm Caspase-3/7 Green flow cytometry assay kit was purchased from Invitrogen (Thermo Fisher Scientific Inc., Waltham, MA, USA).

### 4.3. Generation of Cytokine-Induced Killer (CIK) Cells

CIK cells were generated according to a previously described protocol [60]. Briefly, peripheral blood mononuclear cells (PBMCs) were isolated from the blood of healthy donors (blood bank of the University Hospital Bonn, Bonn, Germany) through gradient centrifugation using Pancoll (Aidenbach, Bavaria, Germany). PBMCs were cultured at 2 × 10^6^/mL in a 75 cm^2^ flask, and 1000 U/mL of IFN-γ (ImmunoTools GmbH, Aidenbach, Bavaria, Germany) was added 2 h later. On the second day, monocytes were removed, and 100 U/mL of IL-1β (ImmunoTools GmbH, Aidenbach, Bavaria, Germany), 600 U/mL of IL-2 (ImmunoTools GmbH, Aidenbach, Bavaria, Germany) and 50 ng/mL of anti-CD3 (Thermo Fisher Scientific, CA, USA) were added. CIK cells were then cultured in RPMI-1640 medium (Pan-Biotech, Aidenbach, Bavaria, Germany), and supplemented with 10% FBS (Sigma-Aldrich Chemie GmbH, Munich, Germany), 2.5% HEPES (Gibco, Thermo Fisher Scientific, Inc.), and 1% penicillin/streptomycin (P/S) (Gibco, Schwerte, Germany) at 37 °C, 5% CO_2_, and humidified atmosphere. Subcultures were obtained every 3 days at 0.5–1 × 10^6^ cells/mL in fresh medium containing 600 U/mL of IL-2. After two weeks of ex vivo expansion, CIK cells were collected for the experiments.

### 4.4. CCK8 Assay

Cell viability was assessed using the Cell Counting Kit-8 (CCK8, Dojindo Molecular Technologies, Inc., Rockville, MD, USA) assay. Raji and BL41 cells were seeded at a density of 1 × 10^4^ cells per well in a U-bottom Nunclon™ 96-well plate and exposed to a range of concentrations of 17-DMAG and ganetespib for 48 h. Subsequently, 10 μL of CCK-8 working solution was added to each well and incubated for 2–4 h. Absorbance at 450 nm and 650 nm was measured using a Fluostar OPTIMA microplate reader (BMG Labtech, Ortenberg, Germany).

### 4.5. Phenotype Expression of CIK Cells

Mature CIK cells (at day 14) were used to confirm their phenotype using flow cytometry. The CIK cells were stained with PE-CD56, FITC-CD3, APC-NKG2D, BV421-CD8, and their corresponding isotype antibodies. 7AAD was used to stain the dead cells. Samples were acquired using flow cytometry (FACS Canto II, BD Biosciences, Heidelberg, Germany).

### 4.6. Cytotoxicity of CIK Cells

Tumor cells (1 × 10^6^) were labeled with 1uM CFSE in 1 mL DPBS and incubated for 15 min at 37 °C in darkness. The cells were then washed twice with 10 mL culture medium to remove any excess CFSE dye. Subsequently, 2 × 10^4^ tumor cells per well were co-cultured with a medium containing either 17-DMAG (0.1 µM) or ganetespib (20 nM) in a 37 °C, 5% CO_2_ incubator for 24 h. Afterward, CIK cells were added while maintaining the E:T ratio of 10:1. To perform NKG2D blocking experiments, CIK cells were pretreated with purified anti-human CD314 (clone 1D11, BioLegend, Koblenz, Germany) or isotype mouse IgG1 κ (10 ug/mL) for 30 min. Following pre-treatment, CIK cells were co-cultured with tumor cells for 20–24 h. Dead cells were then labeled with Hoechst 34580 and analyzed using flow cytometry. The CFSE-labeled cells were identified as tumor cells, whereas the other cells were identified as CIK cells. The formula used to calculate cytotoxicity was described as the following: Cytotoxicity (%) = (CT − TE)/CT × 100. CT: tumor live cells with CFSE. TE denotes live tumor cells with CFSE following treatment with HSP90 inhibitor or/and CIK cells.

### 4.7. MICA/B Expression

Raji and BL41 cells were seeded at a density of 2 × 10^4^ cells per well in 48-well plates with complete medium containing 17-DMAG (0.1 µM) or ganetespib (20 nM) at a total volume of 300 µL per well for 48 h. The cells were then collected and washed twice with DPBS to remove any remaining medium. To assess MICA/B expression, cells were stained with an APC-MICA/B antibody and analyzed using flow cytometry; viable cells were labeled with Hoechst.

### 4.8. The Apoptosis of Burkitt’s Lymphoma Cells

To analyze the apoptosis of BL cells, 1 × 10^6^ Raji and BL41 cells in 1ml DPBS were labeled with 0.5 µM violet dye for marking tumor cells under the conditions of 37 °C in darkness for 15 min. The cells were then washed twice with 10 mL culture medium to remove any excess dye. Subsequently, 2 × 10^4^ cells per well were seeded in 48-well plates (with a total volume of 300 µL) and co-cultured with HSP90 inhibitors (experimental group) or DMSO (control group) for 24 h. The appropriate number of CIK cells were then added to maintain an E:T ratio of 5:1 for 20–24 h. Afterward, the cells were washed with Annexin V buffer and stained with FITC-Annexin V antibody and 7AAD dye for 15 min.

### 4.9. Caspase 3/7 Activity

To label the tumor cells, Raji and BL41 cells (1 × 10^6^) were incubated with 0.5 µM violet dye in 1 mL DPBS at 37 °C for 15 min in an incubator. The cells were washed twice with 10 mL culture medium to eliminate excess dye and then seeded at a density of 1 × 10^4^ cells per well in 96-well plates with or without HSP90 inhibitors for 36 h. Next, CIK cells were added at an E:T ratio of 2.5:1. To evaluate caspase3/7 activity, the CellEvent™ Caspase-3/7 Green Flow Cytometry Assay Kit was utilized to stain the tumor cells with 0.5 μM CellEvent™ Caspase-3/7 Green Detection Reagent and 1 μM SYTOX™ AADvanced™ Dead Cell Stain at room temperature for 1 h and 5 min, respectively. The percentage of activated caspase-3/7 cells was determined using flow cytometry.

### 4.10. Fas Expression

Raji and BL41 cells (2 × 10^4^ cells per well) were seeded in 48-well plates and treated with HSP90 inhibitors at 37 °C and 5% CO_2_ for 48 h. After washing with PBS twice, the cells were stained with PE-CD95 for 30 min. Hoechst 34580 was then added, following which flow cytometry measurements were taken.

### 4.11. Correlation of HSP Genes with BL Patients

The BL dataset was downloaded from the TCGA database (https://portal.gdc.cancer.gov/projects/CGCI-BLGSP (accessed on 26 March 2023), Project ID: CGCI-BLGSP). The log-rank test (Mantel–Haenszel) was used to detect significant differences in patient survival according to HSP expression (low and high expression groups). The cut-off value for each gene was the median expression.

### 4.12. Statistical Analysis

Flow cytometry datasets were analyzed using FlowJo V10.6 (LLC, Ashland, OR, USA). Statistical analyses were performed and figures were prepared using GraphPad Prism software (version 9.0; GraphPad Software, San Diego, CA, USA), including the one-way or two-way analysis of variance (ANOVA) with the Bonferroni test and Student’s *t*-test. Bioinformatics analyses were performed using R statistical software (version 4.1.1). Statistical significance was set at *p* < 0.05. * *p* < 0.05, ** *p* < 0.01, *** *p* < 0.001, **** *p* < 0.0001.

## Data Availability

Not applicable.

## Supplementary Materials

The following supporting information can be downloaded at: https://www.mdpi.com/article/10.3390/ijms241512476/s1.

## Abbreviations

CIK cell: cytokine-induced killer cell; NK cell: natural killer cell; NKT cell: natural killer T cell; NKG2D: natural killer group 2, member D; NKG2DL: NKG2D ligand; MICA/B: major histocompatibility complex class I polypeptide-related sequence A and B; HSP: heat shock protein; 17-DMAG: 17-dimethylaminoethylamino-17-demethoxygeldanamycin; DMSO: dimethyl sulfoxide; BL: Burkitt’s lymphoma; PI3K: phosphoinositide 3-kinase; SYK: spleen tyrosine kinase; TNF: tumor necrosis factor; IFN-γ: interferon-γ; IL-2: interleukin-2; 7AAD: 7-amino-actinomycin D; TCGA: the cancer genome atlas; KM curve: Kaplan–Meier curve; ICI: immune checkpoint inhibitor; and HDAC: histone deacetylase.
